# Biodegradation of Low-Density Polyethylene—LDPE by the Lepidopteran *Galleria Mellonella* Reusing Beekeeping Waste

**DOI:** 10.3389/fbioe.2022.915331

**Published:** 2022-09-09

**Authors:** Orlando Poma, Betty Ricce, Jeyson Beraún, Jackson Edgardo Perez Carpio, Hugo Fernandez, Juan Soria

**Affiliations:** ^1^ Escuela Profesional de Ingeniería Ambiental, Facultad de Ingeniería y Arquitectura, Universidad Peruana Unión, Lima, Perú; ^2^ Escuela Profesional de Ingeniería Ambiental, Facultad de Ingeniería y Arquitectura, Universidad Peruana Unión, Juliaca, Perú; ^3^ Escuela UPG Ingeniería y Arquitectura, Escuela de Posgrado, Universidad Peruana Unión, Lima, Perú

**Keywords:** pollution, plastic, *Galleria mellonella*, LDPE, biodegradation

## Abstract

Plastic pollution is one of the most serious environmental problems of this century because most plastics are single-use, and once their useful life is over, they become pollutants, since their decomposition takes approximately 100–400 years. The objective of this research is to evaluate the efficacy of low-density polyethylene (LDPE) biodegradation by *G. mellonella* in the district of Pangoa, Junín, Peru. For the development of the study, the *G. mellonella* was conditioned in three groups of beekeeping residues (beeswax, balanced diet, and wheat bran); after the conditioning stage, the biodegradation treatment was developed, which consisted of placing the *G. mellonella* in terrariums with the LDPE, the treatments were carried out at three different times (24, 36, and 48 h). To evaluate the efficacy of biodegradation, two analyses were taken into account: the Raman analysis of the low-density polyethylene samples and the weight reduction of the treated LDPE. The results of the Raman analysis indicated that the best treatment was the one applied with *G. mellonella* conditioned with beeswax, obtaining a wavelength intensity of 0.45 μ.a., while the weight reduction of the LDPE indicated that the best results were given at 36 h and conditioned with beeswax with a reduction of 236.3 mg. In conclusion, the use of *G. mellonella* for the biodegradation of low-density polyethylene is effective when it is conditioned with beeswax and the treatment is carried out at 36 h.

## 1 Introduction

Could anyone live without plastic in the 21st century? Well, no, since a large part of the objects we see and use is made of that material (Cáceres, 2017). It has become so ubiquitous that it is hard to believe that it has only been produced on an industrial scale since 1950 (Geyer et al., 2017).

According to the United Nations Environment Program, plastic pollution is one of the serious environmental problems of this century (Abaza, 2012). Estimates show that to date, 8,300 million metric tons of plastic (MT) have been produced, but about half was created in 2004; and of the total plastics produced, 30% is still in use; the rest (6,000 million MT) has become waste (9% recycled, 12% incinerated, and 79% in a landfill or thrown into the environment) (Geyer et al., 2017). With regard to plastic bags, 10 million bags are used in the world every minute and 5 billion a year, of which about 5–13 million tons are dumped into the ocean (Ellen MacArthur Foundation, 2016). With the appearance of the pandemic caused by COVID-19, its use has increased greatly (Flores, 2020).

In South America, plastic pollution has caused the formation of “plastic islands” in the North Pacific Ocean, and 80% of this waste comes from land sources (Elías, 2015), which is why it is vitally important to find adequate treatments and carry out good management of this type of waste (Urdaneta, 2014).

In the same way (Ministerio del Ambiente, 2014), 886 tons of plastic per day is generated in Lima and Callao (Ministerio del Ambiente, 2017). Currently, each citizen uses 30 kg of plastic per year and the total number of plastic bags is around 3,000 million, almost 6 thousand bags per minute (Ministerio del Ambiente, 2017); most of it is single-use and once its useful life is over, it becomes a pollutant, since its decomposition takes approximately 100–400 years (Bombelli et al., 2017). Since 2016, there have been environmental laws and comprehensive management plans to treat this type of waste, in which the ministries of the environment, health, and education; municipalities; and regional governments are involved (Ministerio del Ambiente, 2016a). Incidentally, in 2018, the law that regulates the use of plastic (Ministerio del Ambiente M, 2019) was enacted; however, due to the lack of control and inspection, the non-existence of control measures, and the absence of environmental education among citizens, not much has been achieved so far (Flores, 2020). On the other hand, the Ministry of Environment has lines of research that encourage this type of study in favor of the environment. This study is within this line of research: Component: Solid and Hazardous Waste; Thematic area: Treatment of solid and hazardous waste; Research line: Solid, organic, hazardous, and chemical waste treatment technologies (Ministerio del Ambiente, 2016b).

Low-density polyethylene (LDPE) is a plastic composed of olefin monomers, and it is polymerized at high pressure, highlighting its flexibility and chemical and dielectric resistance: undoubted characteristics that make it useful for the manufacture of bags and industrial packaging, among others items (Martin, 2012). Their great durability and slow biodegradation mean that these synthetic polymers can tolerate the ocean environment for years, decades, and even longer periods (Gutierrez, 2018). This causes marine animals to get trapped by the garbage; birds and other marine creatures consume plastic when they confuse it with their food; and this waste can act as a raft and move some species out of their area (Gutierrez, 2018). The plastic can be reduced in an ecological way with the help of soil bacteria and water availability, but it depends on the type of material that is made, for example, starch-based polymer is degraded by microbes and hydrolytic enzymes, and polymers to petroleum-based polyolefins, degraded through photodegradation (Pathak and Navneet, 2017).

The development of this study proposes an alternative treatment to plastic, as well as the reduction of the volume of plastic waste that arrives at the CEPAP (Pangoa District Sanitary Landfill) in order to increase the useful life of the transitory cells. The district of Pangoa, Satipo, Junín, has a population of 24,939 inhabitants in the urban area alone; the per capita generation of waste is 0.45 kg/person/day; and 4,113.6 tons of waste are generated per year, with plastic being the waste material with the second highest generation: 168.2 tons per year (Municipalidad Distrital de Pangoa, 2019).

That is why the objective of this research is to evaluate the efficiency of polyethylene biodegradation by treating plastic with *G. mellonella* larvae conditioned with three beekeeping residues (beeswax, balanced diet, and wheat bran) and subjected to three different times (24, 36, and 48 h).

## 2 Materials and Methods

The study was carried out in the district of Pangoa, Junín, Peru (latitude: −11.4281, longitude: −74.4881, latitude: 11°25′41″south, longitude: 74°29′17″west), under laboratory conditions.

### 2.1 Materials and Equipment

The materials to be used for the packaging of the *G. mellonella* are the following:

- Balanced diet [to be prepared with wheat bran (150 gr), ground rice (70 gr), puppy biscuits (25 gr), honey (250 gr), and pollen (8 gr)].
  - - Beeswax 3 kg
  - - Wheat bran 3 kg
  - - LDPE bag
- 2 packs of low-density polyethylene bags—PEBD (brand: Plásticos Alfa, material: low-density polyethylene, measurements: 86 mm × 19 mm, color: transparent, quantity: 50 units); each bag weighs 425.25 mg, and they were obtained from the local market.
  - - Pieces of cardboard (to place the eggs).
  - - Clamp (to remove the LDPE sample without altering or damaging it).
  - - Latex gloves (to handle the sample without altering it).
  - - Cooler
  - - Digital multi-function electronic Scale
  - - Thermo-hygrometer BOECO Germany
  - - Photographic camera
  - - Terrarium (glass box with measurements: 15 cm wide × 20 cm long × 10 cm high, covered with metal mesh and tulle fabric).
- One thousand larvae of *G. mellonella* (30 larvae for each treatment).

### 2.2 Conditioning of *G. Mellonella*

**FIGURE 1:**
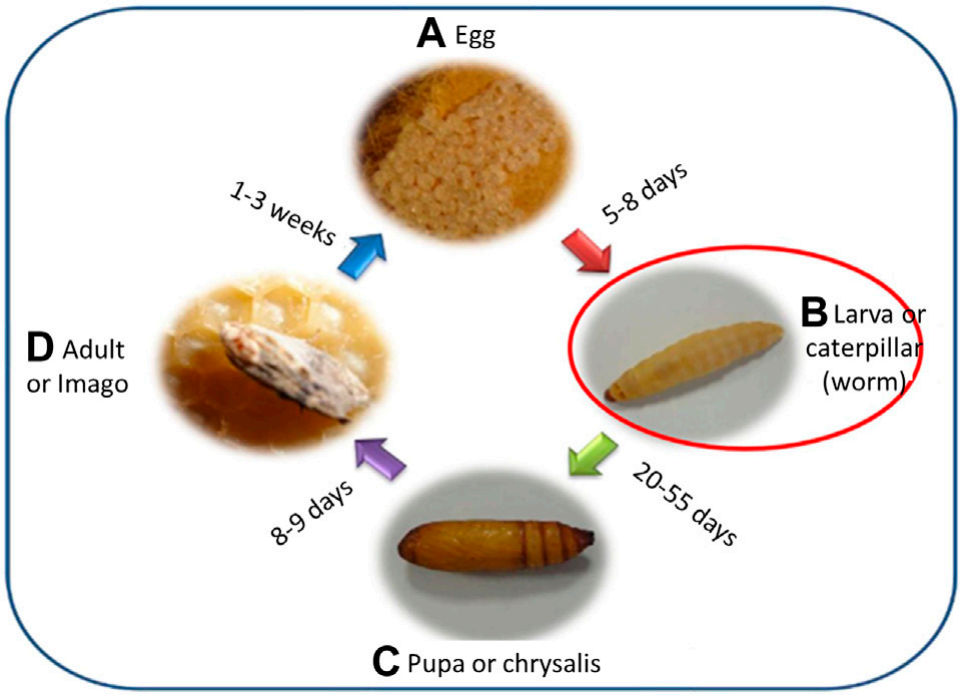
Biological cycle of *G. mellonella*. Source: Lucila Galan (Galán, 2012). **(A)**. Egg, **(B)**. Larva or caterpillar (worm), **(C)**. Pupa or chrysalis, **(D)**. Adult or Imago.

The work area was conditioned, and the wooden boxes for breeding were prepared. The *G. mellonella* was obtained from the company “Apícola Ayni S.A.C.” in the pupal and adult stage, and breeding was carried out in three groups with a different food for each group: A, balanced diet; B, beeswax; and C, wheat bran (Anwar et al., 2014). When *G. mellonella* reaches the larval stage, the stage in which it biodegrades LDPE (see Figure 1), the study begins.

### 2.3 Treatment

The low-density polyethylene-LDPE was previously analyzed in terms of weight and Raman spectroscopic effect to submit them to the treatments and control group. Surgical gloves and sterilized tweezers were used for handling and taking samples to avoid possible contamination of the sample. Then, it was subjected to treatment with *G. mellonella* obtained from the three groups mentioned (A, beeswax; B, balanced diet; and C, wheat bran), developing at three different times (24, 36, and 48 h) with three repetitions for each treatment (Montgomery and Wiley, 2004), and using 30 larvae for each treatment; the treatments were carried out by applying the larvae and adding 5 g of residue in each polyethylene treatment. Finally, the final weighing of the low-density polyethylene PEBD and the Raman spectroscopic effects of each of the treatments were carried out. For the latter, the services of the Mycology and Biotechnology Laboratory of the La Molina National Agrarian University were provided. It should be noted that among all the treatments, only 3 *G. mellonella* larvae died in the treatments: C1, 24 h; C3, 48 h; and A1, 48 h because they were at the beginning of the growth stage, the larval stage, and were just adapting to survival conditions. Polyethylene ingestion did not cause significant growth of *G. mellonella*.

### 2.4 Data Processing

The data obtained were processed for the publication of the results. The statistical design chosen for this study was the 32 factorial design since in this study we worked with 2 factors: A (beekeeping residue) and B (biodegradation time) and each factor had three levels: factor A (beeswax, balanced diet, and wheat bran) and factor B (24, 36, and 48 h); the product of the interaction of the factors with the levels, nine combinations of treatments, were obtained (Montgomery and Wiley, 2004). A normality test was performed by applying the Shapiro–Wilk test, verifying that a normal distribution was fulfilled, and the ANOVA parametric test was applied for the difference in means; these tests were carried out at a confidence level of 95%. The comparison of the degraded sample was carried out by measuring the weight and the Raman effect of the low-density polyethylene before and after the treatment.

## 3 Results

### 3.1 Weight Results of Low-Density Polyethylene

The analysis of the LDPE remnant was carried out due to the fact that readings of the stool samples of the larvae were not obtained; this remnant was evaluated in the vicinity of the holes made in the consumption of the LDPE of the larvae, and said evaluation was carried out in these points since Bombelli et al. (2017) mentioned that one of the beings responsible for the biodegradation of LDPE was the intestinal flora of *G. mellonella*.

Table 1 presents the results of the analysis of variance (ANOVA of 2 factors) applied to the data obtained from the weight reduction of the LDPE applied the treatment. The results of the statistical test indicated that at a confidence level of 95%, the beekeeping residue applied in the breeding of *G. mellonella* has a very marked effect on the weight reduction of the LDPE and that the treatment time also has a very marked effect on the weight reduction of the LDPE; in addition, it indicated that the interaction between the beekeeping residue applied in the breeding of the *G. mellonella* and the treatment time has great significance in the weight reduction of the LDPE.

**TABLE 1:** Result of analysis of variance (ANOVA for 2 factors).

	Df	Sum sq	Mean sf	F value	*p* value
Beekeeping waste	2	0.2534	0.12671	33.84	7.98*10^−7^
Treatment time	2	0.3645	0.18226	48.67	5.49*10^−8^
Interaction between applied beekeeping residue and treatment time	4	0.1602	0.04004	10.69	0.000129
Residuals	18	0.0674	0.00374		

**FIGURE 2:**
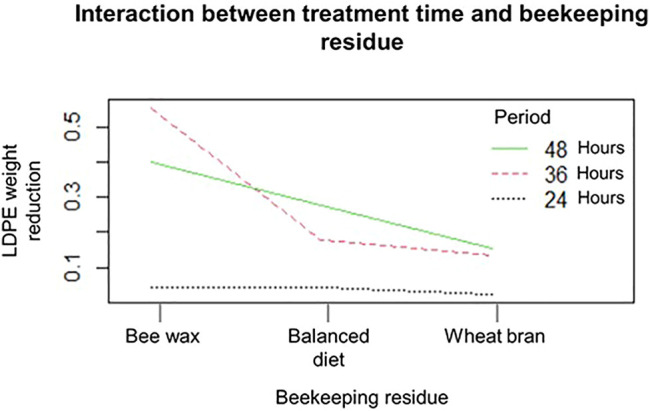
Interaction between treatment time and beekeeping residue in the rearing of *G. mellonella*.

In Figure 2, the interaction between the treatment time and the effect of the beekeeping residue applied in the breeding of *G. mellonella* can be seen. This graph indicates that for the larva conditioned with beeswax, the best time of treatment is 36 h, obtaining a greater average weight reduction of LDPE of 55.6% (263.3 mg); for the larva conditioned with the balanced diet and for the larva conditioned with wheat bran, the best treatment time is 48 h, obtaining an average LDPE weight reduction of 27.8% (118.1 mg) and 15.6% (66.2 mg), respectively.

**FIGURE 3:**
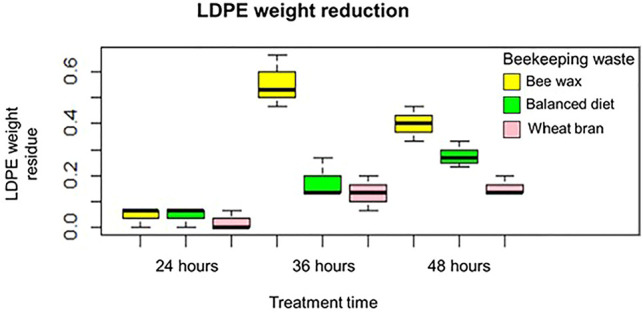
LDPE weight reduction.

In Figure 3, we can observe the weight reduction of the LDPE when subjected to the treatment; of the nine applied treatments, it is observed that the one with the greatest weight reduction is the treatment applied with the larva conditioned with beeswax at a treatment time of 36 h, with an average weight reduction result of LDPE of 55.6% (263.3 mg). In addition to this, it is observed that the second best result is obtained with the larva conditioned with beeswax at a treatment time of 48 h with an average LDPE weight reduction result of 40.0% (170.1 mg). These results indicate that the LDPE subjected to treatment with the larva conditioned with beeswax obtains better results compared to those conditioned with a balanced diet and wheat bran; in addition, the treatment of the larva conditioned with beeswax obtains better results with a treatment time of 36 h.

### 3.2 Low-Density Polyethylene Raman Analysis Result

#### 3.2.1 Quantitative Analysis

Table 2 presents the results of the analysis of variance (ANOVA) applied to the data obtained from the wavelength intensity of the spectrum of the LDPE Raman analysis; the results of the statistical test indicated that at a confidence level of 95%, the beekeeping residue applied in the breeding of *G. mellonella* has a very marked effect on the reduction of the intensity of the wavelength of the LDPE Raman spectrum and that the treatment time has no marked effect on the reduction of the wavelength intensity of the LDPE Raman spectrum.

**TABLE 2:** Results of the analysis of variance (ANOVA) of the wavelength intensity of the Raman spectrum.

	Df	Sum sq	Mean sf	F value	*p* value
Beekeeping waste	3	0.4276	0.14253	5.068	0.00587
Treatment time	2	0.1707	0.08535	3.035	0.06305
Residuals	30	0.8438	0.02813		

**FIGURE 4:**
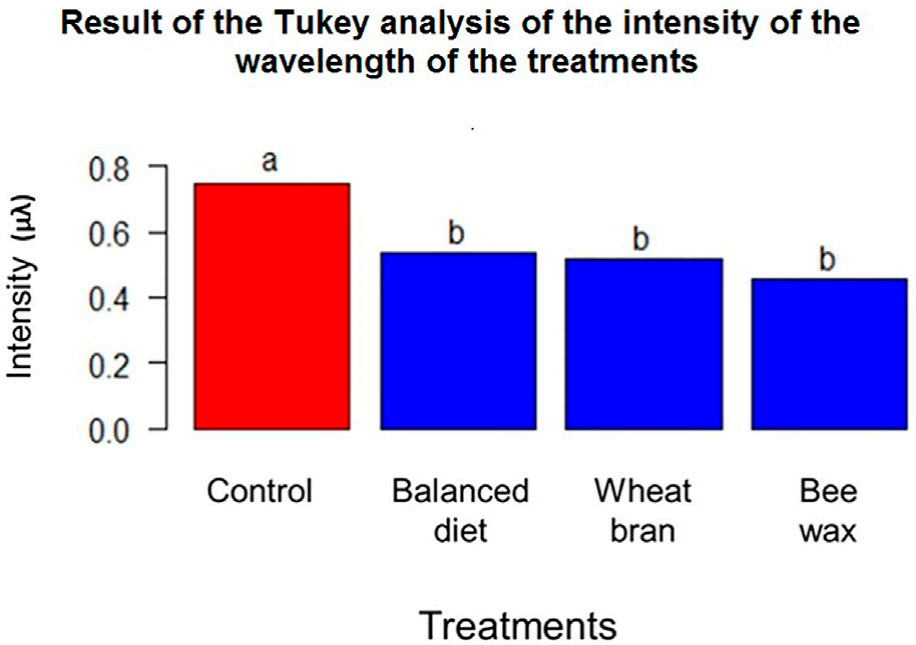
Result of the Tukey analysis of the intensity of the wavelength of the treatments.

In Figure 4, the results of the Tukey analysis of the wavelength intensity of the LDPE Raman spectrum highlighted with the beekeeping residues and with the treatment control are presented; the results of the statistical test indicated that at a confidence level of 95%, the wavelength intensity of the control is the only result that differs from the averages of beekeeping waste, thus indicating that there is a significance in the reduction of the wavelength intensity applying this treatment.

#### 3.2.2 Qualitative Analysis

**FIGURE 5:**
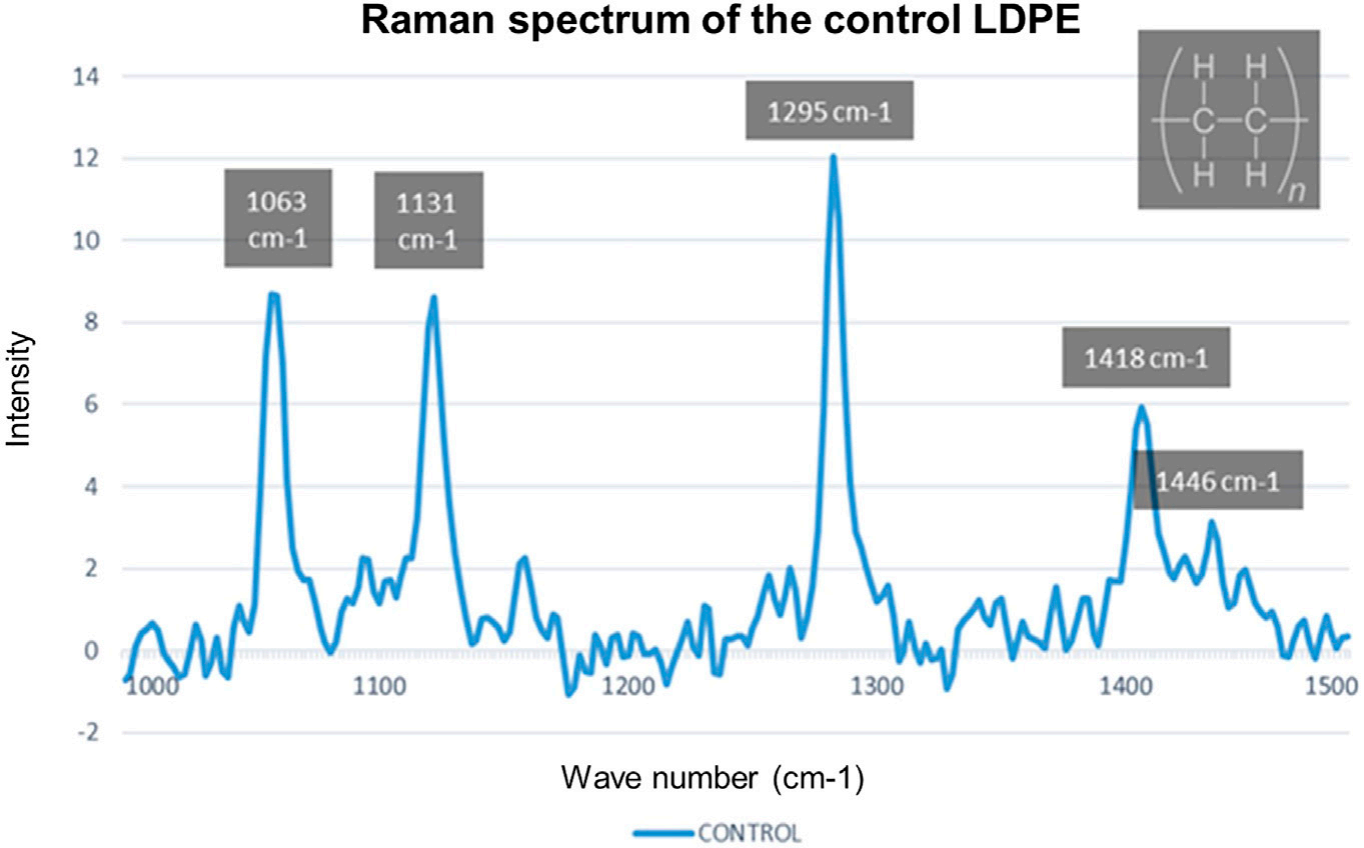
Raman spectrum of the control LDPE.

In Figure 5, the Raman spectrum of the control low-density polyethylene (LDPE) is shown, highlighting the most significant peaks, and Table 3 details the functional group obtained in the peaks. The Raman bands of the spectrum for the crystal chain (1,063, 1,131, 1,295, and 1,418 cm^−1^) were taken, and these bands were used as a standard to compare the variations of the LDPE crystallinity (Kida et al., 2016).

**TABLE 3:** Wave number and functional group [the values were assigned by comparing with the spectra reported by Kida et al. (2016)].

Wave number (cm^−1^)	Functional group
1,063	C-C
1,131	C-C
1,295	CH2
1,418	CH2
1,446	CH2

The Raman spectra of the treatments carried out were compared with the control sample of the LDPE, and it was found that 74% of the treatments obtained a reduction in the intensity observed in the wavelength 1,063 cm^−1^; 56% of the treatments, in the wavelength 1,131 cm^−1^; 96% of the treatments, at the wavelength 1,295 cm^−1^; and 100% of the treatments, at the wave number 1,418 cm^−1^. In this sense, the intensity reduction in these Raman bands indicated a decrease in the crystallinity of LDPE as a result of the treatment applied to the samples, in addition to a decrease in the functional groups C-H and CH2. The breaking of the C-C bonds implies a level of degradation of the low-density polyethylene molecules—LDPE. Therefore, there is a decrease in polyethylene as a contaminating factor due to its interaction with *G. mellonella*.

**FIGURE 6:**
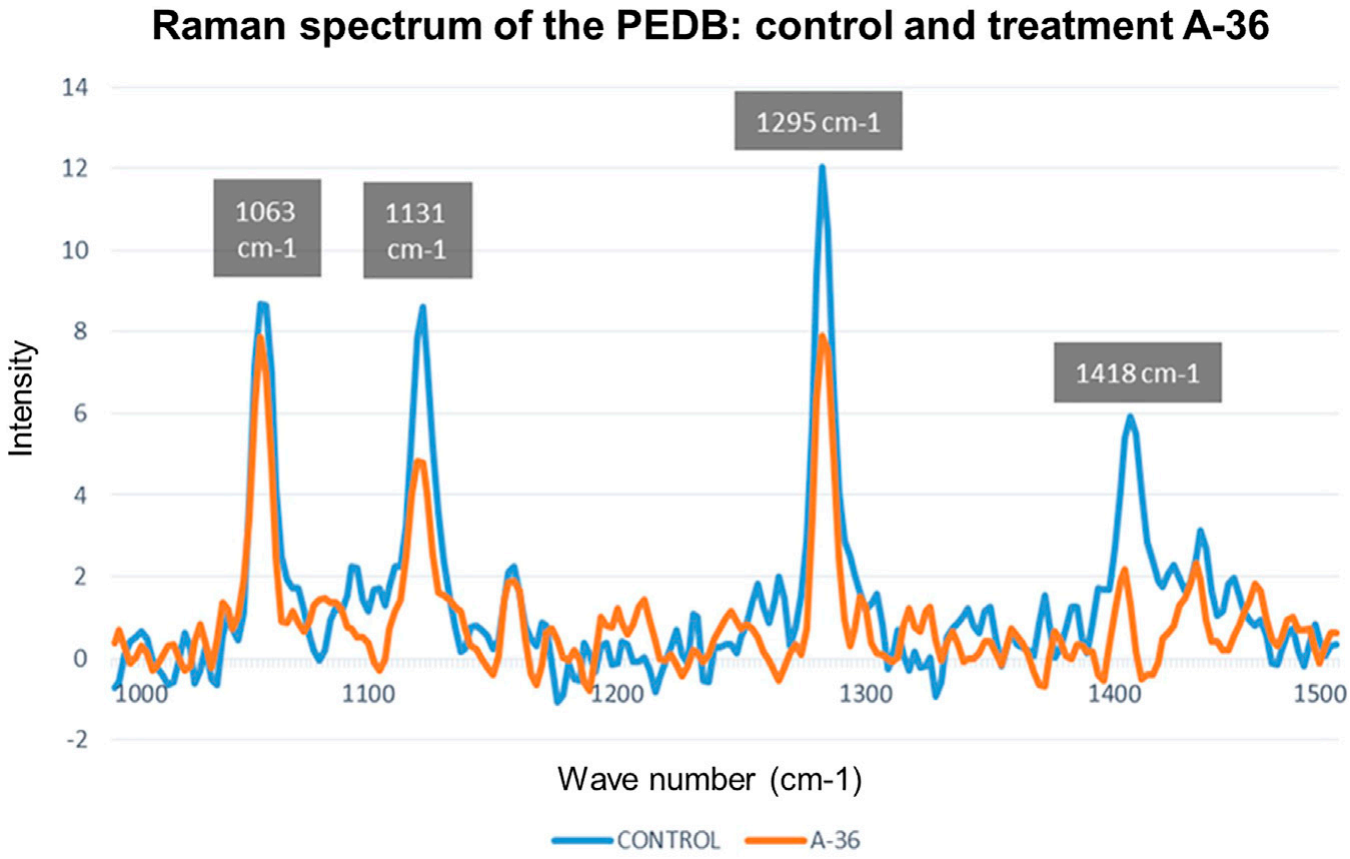
Raman spectrum to contrast the difference between the LDPE control and the treatment A-36 h.

Figure 6 shows the Raman spectrum of the control LDPE and the A-36 h treatment since it is the treatment that obtained the best results.

### 3.3 Result of the Physical Change of the Low-Density Polyethylene

**FIGURE 7:**
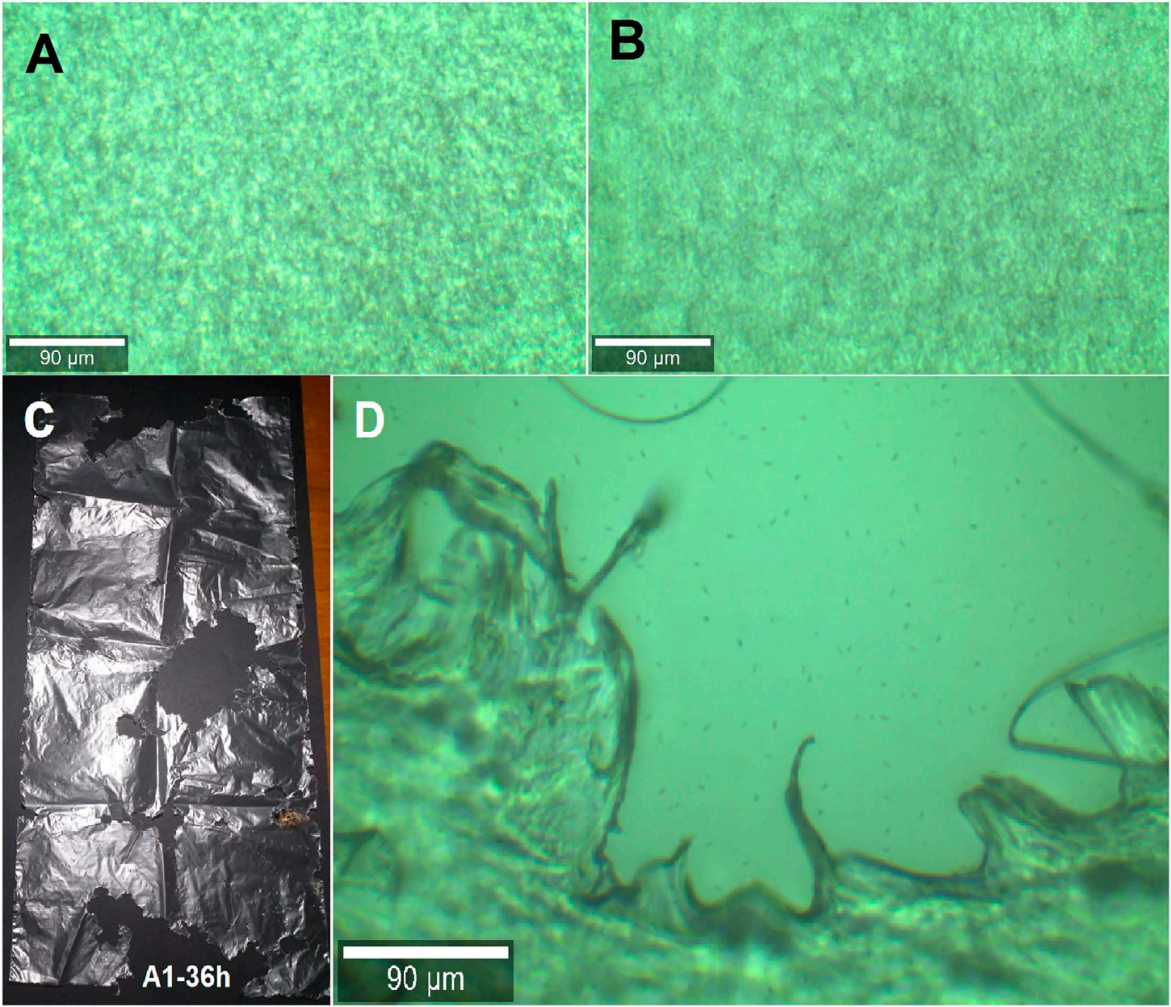
Result of the physical change of the LDPE. In **(A)**, the optical micrograph of the control sample is observed and in **(B)**, the A1-36 h treatment; **(C)** shows the evidence of LDPE consumption of the A1-36 h treatment with a normal scale view, and **(D)** presents a light micrograph view of sample A1-36 h-a2.

Figure 7 shows the differences between the control sample (Figure 7A) and the A1-36 h treatment of low-density polyethylene—LDPE (Figure 7B), showing a slight change in brightness and the appearance of some cracks; this was analyzed in 100% of the sample and with a level of 90 μm, a ×20 Zeiss EC Epiplan objective (numerical aperture 0.4) with a white light LED for Köhler illumination was used; all micrographs were exported in TIF format with dimensions of 1,024 × 653 pixels; the activity that the *G. mellonella* larvae had on the LDPE was also observed with the naked eye (Figure 7C), an optical view made at the edges of the holes left by the *G. mellonella* larvae in the low-density polyethylene sample. LDPE (Figure 7D) was analyzed in the region “a” with a level of 23.36 µm.

## 4 Discussion

The analysis of the LDPE remnant was carried out due to the fact that readings of the stool samples of the larvae were not obtained, this remnant was evaluated in the vicinity of the holes made in the consumption of the LDPE of the larvae, and the said evaluation was carried out in these points since Bombelli et al. (2017) mentioned that one of those items responsible for the biodegradation of LDPE is the intestinal flora of *G. mellonella*. In the present investigation, it was found that 30 larvae of the lepidoptera *G. mellonella* conditioned with “beeswax” biodegraded 263.3 mg (55.6%) of low-density polyethylene—LDPE—in 36 h at an average temperature of 25°C and a relative humidity average of 83%; it belongs to Treatment A1-36.

One of the ways to assess biodegradation efficiency is the amount of LDPE weight lost; in other words, how many milligrams of LDPE did *G. mellonella* consume? Bombelli et al. (2017) mention that 100 *G. mellonella* larvae are capable of biodegrading 92 mg of polyethylene in 12 h. Although we extended the time to 36 h, but applied only 30 larvae, we obtained a greater amount of biodegraded LDPE; in other words, 263.3 mg of biodegraded LDPE, which is higher, even if we double Bombelli’s 92 mg.

Alkassab et al. (2020), found that 10 larvae of *G. mellonella* degrade 0.0210 mg in 12 h at a temperature of 25°C. In our study, we obtained a degradation of 263.3 mg, and it is very high; this is because we increase the time and the number of larvae. However, we kept the same temperature of 25°C.

Márquez (Mandal and Vishwakarma 2016) showed that 480 *G. mellonella* larvae were conditioned at a temperature of 27°C and 70% relative humidity biodegraded 0.173 mg of polyethylene in 7 days. In this study, we see that the results are below the other results obtained in our study despite the fact that the treatment time was longer. Mandal and Vishwakarma (2016) mention that there is no relationship between relative humidity and larval growth. Yang (Yang et al., 2014)- Rivas (Rivas, 2020) reported that the American Periplaneta cockroach, conditioned at a temperature of 35°C and 60% relative humidity, biodegrades 0.6 mg of plastic bag (LDPE) in 7 days, obtaining an efficiency of 7.6%. This is very similar to *G. mellonella*, as both are active at night and like the same conditions of temperature and relative humidity.

Nevertheless, in our research, it was found that the larvae of *G. mellonella* biodegraded more low-density polyethylene at 36 h than at 48 h, due to the fact that at 36 h, a great concentration of the larvae was observed in the central part of the box (which has a reduced size of 10 cm × 20 cm) and they fed, very different from the activity at 48 h where the dispersion of the larvae was observed and they did not consume the LDPE in the same way, that is why Kwadha (Kwadha et al., 2017) mentions that the larvae feed more when they are together. On the other hand, the larvae that were at 36 h were more active and those that were at 48 h were not, since they were already beginning to pupate (Rodriguez, 2015).

Compared to these studies, it is shown that our research obtained better results of LDPE biodegradation in terms of weight loss, and taking into account the time, it was also relatively short. On the other hand, the temperature played an important role in the biodegradation of LDPE. Alkassab et al. (2020) worked with *G. mellonella* larvae at 25°C and 35°C, showing that the optimal temperature to carry out biodegradation is 25°C. Márquez (Mandal and Vishwakarma 2016) also worked with the same type of larvae at a temperature of 27°C. Rodríguez (Rodriguez, 2015) mentions that the larvae of *G. mellonella* have a normal development when they are at a temperature of 25°C and their lifetime ranges between 28 days. But if the temperature is too low, their activity becomes slow and their lifetime is prolonged; on the contrary, if the temperature is too high, their activity is accelerated and their lifetime is greatly shortened. For this reason, in our research, we work at a temperature of 25°C.

Regarding the feeding and conditioning of *G. mellonella*, we apply beeswax, a balanced diet, and wheat bran, since Salas (Salas, 2015) considers that the best diet to raise *G. mellonella* contains wheat bran, sugar, and honey. Rodríguez (Rodriguez, 2015) also includes in his diet: wheat bran, rice, croquettes of can, sugar honey, and honey; that is why we opted for these beekeeping residues as food to raise and condition *G. mellonella*. Márquez (Mandal and Vishwakarma 2016) found that treatments that include beeswax have a higher consumption of plastics; this is similar to our work, since the treatment with beeswax obtained better results because under natural conditions, these larvae feed on beeswax and your body is already used to it, you do not need to undergo an adaptation process that generates delayed development or slow activity (Kwadha et al., 2017). It should be noted that the contaminants do not show detectable impacts on beeswax (Alkassab et al., 2020). It should be noted that the contaminants do not show detectable impacts on beeswax (Alkassab et al., 2020), so the use of this residue to condition *G. mellonella* does not affect its growth. Moreover, it was observed that the growth of *G. mellonella* was not affected by the intake of low-density polyethylene-LDPE due to the fact that the composition and chemical structure of LDPE are very similar to those of beeswax (Bombelli et al., 2017).

Finally, Coreño (Coreño and Méndez, 2010) indicates that the crystallinity of polymers is a fundamental part of the solubility and permeability of said elements, that is to say, the reduction of the crystallinity contributes to increasing the solubility and permeability; it also indicates that a reduction in crystallinity reduces the density of the polymer and therefore its resistance.

## 5 Conclusion

With the development of this research work, the following results are obtained:

- The comparison of the Raman spectra indicates that there is a reduction in the crystallinity of LDPE, finding that *G. mellonella* conditioned with beeswax obtains better results with an average wavelength intensity of 0.45 µ.a.
- Beeswax is the residue of beekeeping conditioned with *G. mellonella*, and it obtains better results compared to the other applied treatments, which obtains an average weight reduction of 141.8 mg (33.3% reduction).
- The treatment developed at 36 h is the one that obtains the best results with an average weight reduction of low-density polyethylene of 122.9 mg (28.9% reduction).

In general, it is concluded that the most effective treatment is the one given with *G. mellonella* conditioned with beeswax and with a treatment time of 36 h, since the best results were obtained with an average weight reduction of low-density polyethylene of 236.3 mg (55.6% reduction) in addition to obtaining the best wavelength intensity of 0.45 µ.a.

## Data Availability

The original contributions presented in the study are included in the article/Supplementary Materials; further inquiries can be directed to the corresponding authors.
