# Supplementary material for: Biodegradation of Low-Density Polyethylene—LDPE by the Lepidopteran Galleria Mellonella Reusing Beekeeping Waste
Source: Front Bioeng Biotechnol. 2022 Sep 9;10:915331. doi: 10.3389/fbioe.2022.915331 (PMC9535396; doi:10.3389/fbioe.2022.915331)
Supplement: Supplementary file 1 [file DataSheet1.PDF]

Supplementary Material

1 Supplementary Data

Tables

Tabla 1. Result of analysis of variance (ANOVA for 2 factors)

	Df	Sum Sq	Mean Sf	F value	P value
Beekeeping waste	2	0.2534	0.12671	33.84	$7.98*10^{-7}$
Treatment time	2	0.3645	0.18226	48.67	$5.49*10^{-8}$
Interaction between applied beekeeping residue and treatment time	4	0.1602	0.04004	10.69	0.000129
Residuals	18	0.0674	0.00374		

Table 2 Table 2. Results of the analysis of variance (ANOVA) of the wavelength intensity of the RAMAN spectrum

	Df	Sum Sq	Mean Sf	F value	P value
Beekeeping Waste	3	0.4276	0.14253	5.068	0.00587
Treatment time	2	0.1707	0.08535	3.035	0.06305
Residuals	30	0.8438	0.02813		

Table 3. Wave number and functional group (the values were assigned by comparing with the spectra reported by Kida, Hiejia, Nitta (19))

Wave Number (cm-1)	Functional group
1063	C-C
1131	C-C
1295	CH2
1418	CH2
1446	CH2

2

LDPE weight data and Raman analyzes are in Excel, and will be provided with this supplementary material..

3 Figures

3.1 Statistical analysis

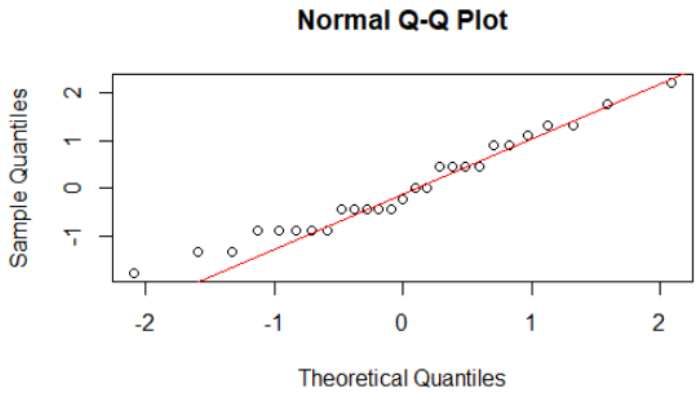

Figure 1. Graph of normality of the results of the LDPE weight reduction

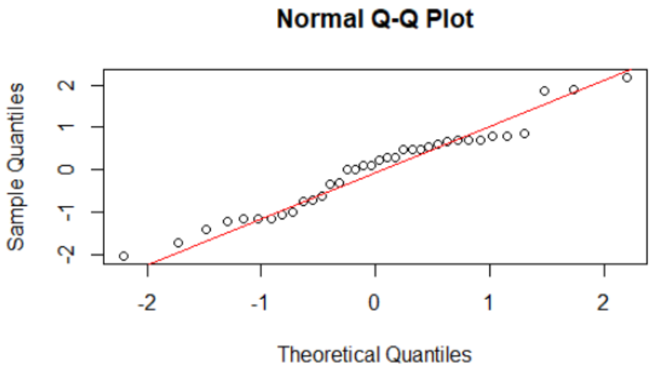

Figura 2. Normality Plot of Wavelength Intensity Results

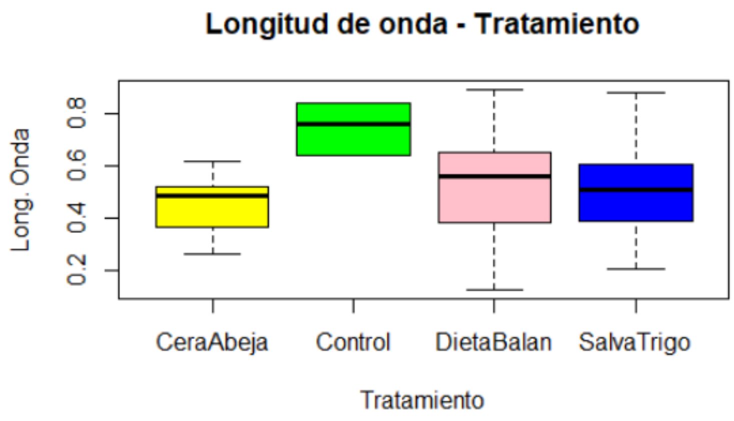

Figura 3. Plot of wavelength intensity averages, highlighted with beekeeping residue used to condition Galleria mellonella

3.2 Fieldwork

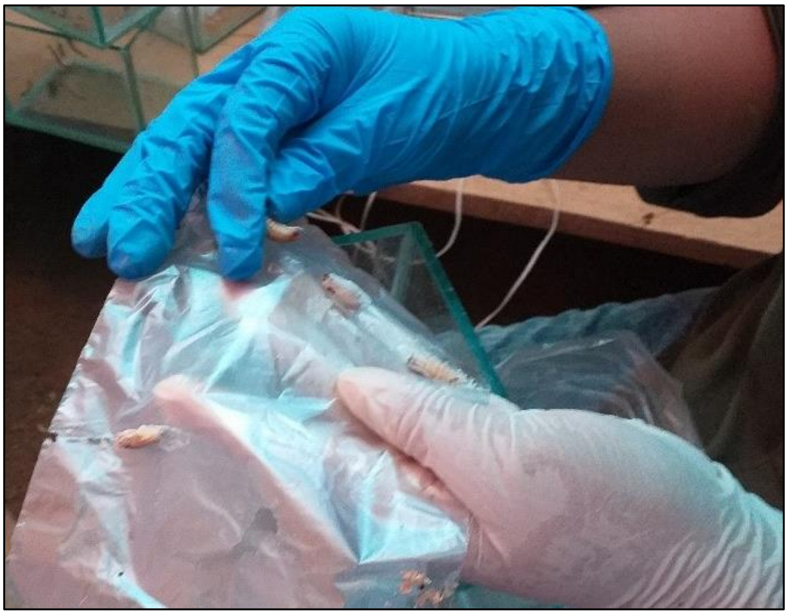

Figure 4. Removal of Galleria mellonella larvae to extract the LDPE sample

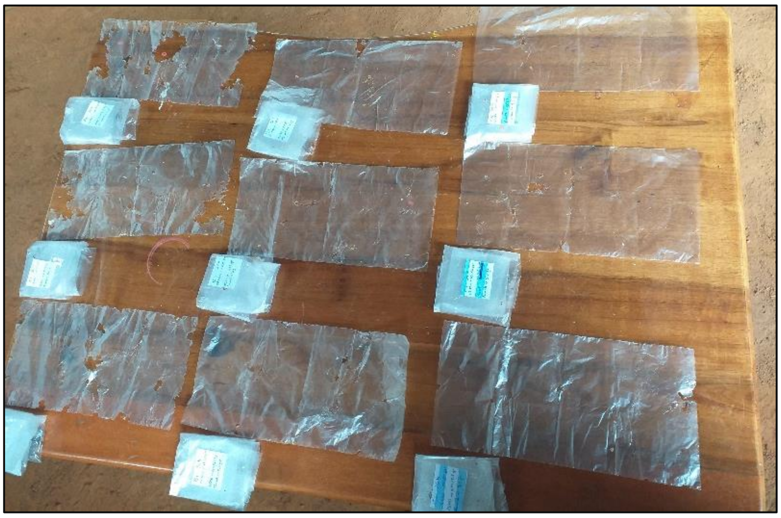

Figure 6. Evidence of the treatments with the 3 types of foods and the 3 repetitions

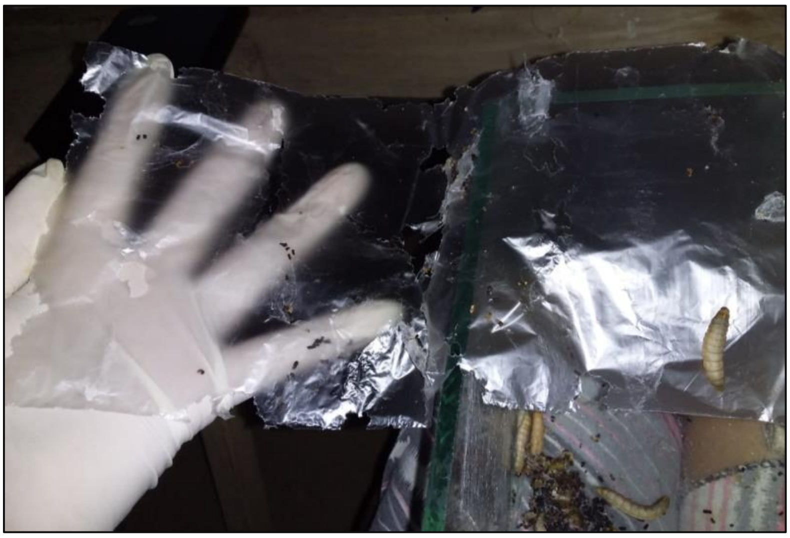

Figure 7. Evidence of LDPE consumption by Galleria mellonella larvae

Laboratorio de Micología y Biotecnología
Universidad Nacional Agraria La Molina
Fono 51-1-6147800 Ext.463
Av. La Molina s/n Lima 12, Perú

La Molina, 01 de diciembre de 2021

Informe descriptivo de medidas por Microscopía Raman Confocal y Microscopía Óptica

Detalles generales del servicio

Tipo de muestras:	Bolsas de polietileno de baja densidad (PEBD)
Número de muestras:	30
Institución:	Universidad Peruana Unión
Persona de contacto:	Betty Ricce / Jeyson Beraun

Detalles instrumentales

Equipo:	Microscopio Raman confocal con AFM
Modelo:	alpha300RA
Marca:	WITec GmbH
Modo de medida:	Medida puntual de espectros Raman y toma de micrografías ópticas.

Las muestras analizadas fueron 30 bolsas PEBD cuya denominación se detalla en la Tabla 1.

1. Microscopía Raman Confocal

Las condiciones de medida de los espectros Raman fueron las siguientes:

Detector:	Cámara CCD
Ancho:	1650 píxeles
Alto:	200 píxeles
Temperatura:	-61 °C
Espectrómetro:	UHTS300S_GREEN_NIR
Longitud de onda del láser:	784.942 nm
Potencia del láser:	75 mW
Rejilla de difracción:	300 g/mm (BLZ=750nm)
Centro espectral:	1628.473 rel. 1/cm

Figure 8. Descriptive report of measurements by Raman Confocal Microscopy and Optical Microscopy of the Mycology and Biotechnology laboratory of the Universidad Nacional Agraria la Molina (Part 1)..

frontiers

Laboratorio de Micología y Biotecnología
Universidad Nacional Agraria La Molina
Fono 51-1-6147800 Ext.463
Av. La Molina s/n Lima 12, Perú

Objetiva: Zeiss EC Epiplan 20x / 0.4
Número de acumulaciones: 30
Tiempo de integración: 2.0 s

Grupo		ID
A	24 h	1
		2
		3
	36 h	1
		2
		3
	48 h	1
		2
		3
B	24 h	1
		2
		3
	36 h	1
		2
		3
	48 h	1
		2
		3
C	24 h	1
		2
		3
	36 h	1
		2
		3
	48 h	1
		2
		3
Control		1
		2
		3

Tabla 1. Grupos de muestras analizadas por microscopía Raman confocal y microscopía óptica. Se analizaron tres muestras idénticas de cada grupo.

Figura 9. Descriptive report of measurements by Raman Confocal Microscopy and Optical Microscopy of the Mycology and Biotechnology laboratory of the Universidad Nacional Agraria la Molina (Part 2).

Laboratorio de Micología y Biotecnología
Universidad Nacional Agraria La Molina
Fono 51-1-6147800 Ext.463
Av. La Molina s/n Lima 12, Perú

1.1. Procedimiento

Las bolsas de PEBD fueron dobladas de tal forma que se acumularon 16 capas y se colocaron entre dos láminas de vidrio portaobjetos, con el fin de tener un mayor espesor de material que arroje una mejor señal (mayor relación señal/ruido). Se fijó la potencia del láser a 75 mW, se midieron y promediaron 30 espectros Raman de 2 segundos cada uno resultando un espectro característico por muestra. Debido a que las láminas de vidrio portaobjetos también arrojan señal Raman formando lomas amorfas en el espectro, éstas se midieron por separado y se restaron a los espectros de las muestras medidas. Asimismo, se realizó el procesamiento de los espectros que consistió en: *i)* Suprimir los picos debido a rayos cósmicos que impactan en el detector CCD; *ii)* Suavizar los espectros; *iii)* Retirar el ruido de fondo (*background*). El espectro resultante se exportó en formato ASCII de dos columnas (Figura 1).

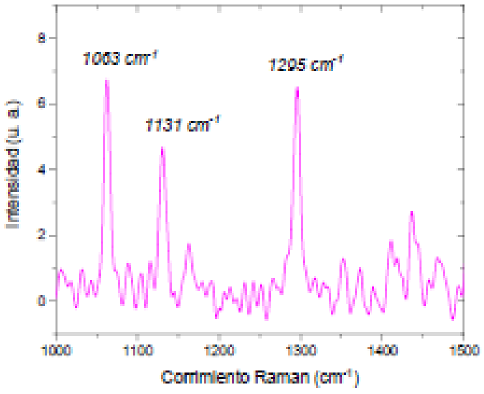

Figura 1. Espectro Raman de la muestra A1-24h.

Por cada una de las 30 muestras, se procesaron y exportaron 2 archivos detallados en la Tabla 2.

Figure 10. Descriptive report of measurements by Raman Confocal Microscopy and Optical Microscopy of the Mycology and Biotechnology laboratory of the Universidad Nacional Agraria la Molina (Part 3).

Laboratorio de Micología y Biotecnología
Universidad Nacional Agraria La Molina
Fono 51-1-6147800 Ext.463
Av. La Molina s/n Lima 12, Perú

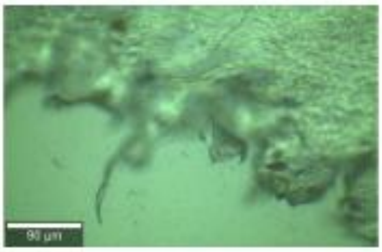

Figura 2. Micrografía óptica de la muestra A2-48h de la región "a" con un nivel de 46.72 µm. Esta imagen fue denominada "A2-48h-a3.tif".

3. Conclusiones

Se pudo notar que, en las muestras en estudio, los picos Raman característicos (1063, 1131, 1295 cm^{-1} aprox.) el ancho a media altura varió con respecto a las muestras control. Por ejemplo, las muestras del grupo Control cuentan con un ancho a media altura de 9.53 ± 1.06 cm^{-1} mientras que el grupo A-48h cuenta con un ancho a media altura de 10.03 ± 2.10 cm^{-1}, lo que indicaría una ligera disminución en la cristalinidad del PEBD producto del tratamiento aplicado a las muestras. Por otro lado, se observaron cambios en las intensidades relativas de los picos antes mencionados, lo que indicaría la reducción de ciertos modos vibracionales en el material en estudio.

Adjunto a este informe descriptivo se envía la carpeta "Medidas UPeU.rar" conteniendo todos los archivos anteriormente descritos.

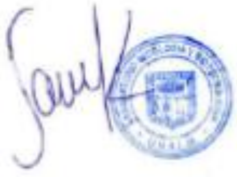

Ilanit Samolski Klein, Ph.D

Figura 11. Descriptive report of measurements by Raman Confocal Microscopy and Optical Microscopy of the Mycology and Biotechnology laboratory of the Universidad Nacional Agraria la Molina (Part 4).

Poma Porras, Orlando; Ricce Maldonado, Betty Ullawda; Beraun, Jeyson; Perez, JPC; Fernandez, Hugo; Soria Quijaite, Juan Jesus (2022), "Biodegradación de polietileno de baja densidad - PEBD - Ricce at al", Mendeley Data, V1, doi: 10.17632/2rgtw557ph.1

CC BY 4.0You can share, copy and modify this dataset so long as you give appropriate credit, provide a link to the CC BY license, and indicate if changes were made, but you may not do so in a way that suggests the rights holder has endorsed you or your use of the dataset. Note that further permission may be required for any content within the dataset that is identified as belonging to a third party.

Reserved DOI: 10.17632/2rgtw557ph.1

Suplementary data
In Mendeley data.
Poma Porras, Orlando; Ricce Maldonado, Betty Ullawda; Beraun, Jeyson; Perez, JPC; Fernandez, Hugo; Soria Quijaite, Juan Jesus (2022), "Biodegradación de polietileno de baja densidad - PEBD - Ricce at al", Mendeley Data, V1, doi: 10.17632/2rgtw557ph.1
